# Relevance of Fc Gamma Receptor Polymorphisms in Cancer Therapy With Monoclonal Antibodies

**DOI:** 10.3389/fonc.2022.926289

**Published:** 2022-06-24

**Authors:** Juan J. Mata-Molanes, Joseba Rebollo-Liceaga, Elena Mª Martínez-Navarro, Ramón González Manzano, Antonio Brugarolas, Manel Juan, Manuel Sureda

**Affiliations:** ^1^ Oncology Platform, Hospital Quirónsalud Torrevieja, Alicante, Spain; ^2^ Department of Immunology, Hospital Clínic de Barcelona, Barcelona, Spain

**Keywords:** cancer immunotherapy, Fc gamma receptor (FcγR), immune checkpoint inhibitors, monoclonal Abs, polymorphisms

## Abstract

Therapeutic monoclonal antibodies (mAbs), including immune checkpoint inhibitors (ICIs), are an important breakthrough for the treatment of cancer and have dramatically changed clinical outcomes in a wide variety of tumours. However, clinical response varies among patients receiving mAb-based treatment, so it is necessary to search for predictive biomarkers of response to identify the patients who will derive the greatest therapeutic benefit. The interaction of mAbs with Fc gamma receptors (FcγR) expressed by innate immune cells is essential for antibody-dependent cellular cytotoxicity (ADCC) and this binding is often critical for their *in vivo* efficacy. FcγRIIa (H131R) and FcγRIIIa (V158F) polymorphisms have been reported to correlate with response to therapeutic mAbs. These polymorphisms play a major role in the affinity of mAb receptors and, therefore, can exert a profound impact on antitumor response in these therapies. Furthermore, recent reports have revealed potential mechanisms of ICIs to modulate myeloid subset composition within the tumour microenvironment through FcγR-binding, optimizing their anti-tumour activity. The purpose of this review is to highlight the clinical contribution of FcγR polymorphisms to predict response to mAbs in cancer patients.

## Introduction

Over the last three decades, the number of therapeutic monoclonal antibodies (mAbs) in clinical use has increased exponentially. During this period, over 80 mAbs have received marketing approval for treating cancer, autoimmune diseases, and infectious diseases by regulatory agencies (1). MAb-based treatment of cancer has been established as a therapeutic strategy for several hematologic and solid tumours, including those that target tumour antigens, anti-human epidermal growth factor receptor 2 (HER2) (e.g. trastuzumab), anti-CD20 (e.g. rituximab) and anti-epidermal growth factor receptor (EGFR) (e.g. cetuximab). More recently, immunomodulatory mAbs that target immune system regulatory molecules have emerged. Immune checkpoint inhibitors (ICIs), including anti-cytotoxic T lymphocyte-associated antigen-4 (CTLA-4) (e.g. ipilimumab), anti-programmed cell death 1 (PD-1) (e.g. nivolumab, pembrolizumab, cemiplimab) and anti-PD ligand 1 (PD-L1) (e.g. durvalumab, avelumab, atezolizumab) have been approved by regulatory agencies in different indications (2). Despite the promising anti-cancer activity shown by therapeutic mAbs, a considerable fraction of patients do not respond to treatment and could even develop mAb-mediated toxicity (3, 4).The search for robust biomarkers to predict response, resistance, or toxicity to these novel therapies is, therefore, mandatory and understanding the mechanisms of action of mAbs is of critical importance.

Therapeutic mAbs belong to the immunoglobulin (Ig) G class of molecules. The anti-tumour activity of these mAbs can be exerted through crystalline fragment (Fc) gamma receptor (FcγR)-independent and FcγR-dependent mechanisms. Tumoricidal effects *in vivo* by FcγR-independent mechanisms include activation of signalling cascades that induce cellular apoptosis through antigen-binding fragment (Fab´)_2_-mediated cross-linking of target molecules and/or signalling inhibition through ligand blockade. FcγR-dependent mechanisms of action include activation of components of the classical pathway of complement and/or recruitment of cytotoxic or phagocytic innate effector cells with FcγR, such as natural killer (NK) or macrophages (5). Antibody-dependent cell-mediated cytotoxicity (ADCC) is defined as the immune mechanism through which Fc-receptor-bearing effector cells can kill target cells that have antigen-antibody complexes on their surface after Fc-FcγR binding. Preclinical studies have demonstrated that recruitment of immune effector cells is essential for ADCC in tumour microenvironment (TME) (5). Antibody-dependent cellular phagocytosis (ADCP) is another important Fc-mediated mechanism of action by which phagocytic cells such as macrophages, monocytes, or neutrophils, contribute to antitumor potency of mAbs. For example, trastuzumab and rituximab have been shown to rely on the activation of FcγRs for efficient tumor killing in breast cancer and lymphoma preclinical models (6, 7). Thus, anti-cancer mAbs can cause different effects inside and outside the TME such as oncogenic pathway blockade, anti-angiogenesis, modulation of immune response against tumour cells or elimination of tumour cells by ADCC or ADCP [reviewed by ref. (8)]. The relevance of the affinity of the Fc-FcγR interaction during ADCC or ADCP has been suggested by preliminary data that show that FcγR genetic variants can significantly influence it (9). Single nucleotide polymorphisms (SNPs) of FcγR can alter ligand binding (e.g. by changing the affinity of FcγR for a particular IgG subclass), affect receptor function or modify its level of expression, and directly impact the effectiveness of immune response for mAb-based regimens in cancer patients (10).

This review focuses on the possible impact of FcγR genetic polymorphisms in a clinical setting and their future contribution as biomarkers of response to therapeutic mAbs. **Table 1** summarizes the most relevant clinical studies published to date. This work was based on a comprehensive search for relevant studies in the PubMed database including the keywords “Fc gamma receptor polymorphisms” AND “Clinical trials” OR “Experimental data” (the last search update was on 18 May 2022).

**Table 1:** Associations between the FcγRIIa and FcγRIIIa polymorphisms and clinical benefit.

mAb	FcγRIIa-131 Genotype	FcγRIIIa-158 Genotype	Cancer Disease	Total Patients	Clinical Association	Study Reference
Rituximab	–	V/V	FL	49	Higher RR	Cartron et al. (11)
Rituximab	H/H	V/V	FL	87	Higher RR	Weng et al. (12)
Rituximab	–	V/V	FL and MCL	171	Higher EFS	Ghielmini et al. (13)
Rituximab	–	V/V	FL	66	Longer OS	Persky et al. (14)
Rituximab	–	V carriers	DLBCL	113	Higher RR and CR	Kim et al. (15)
Rituximab	–	V carriers	DLBCL	34	Higher RR Longer OS	Zhang et al. (16)
Rituximab	–	V carriers	NHL	1050	Higher CR rate (in Asian patients)	Liu et al. (17)
Daratumumab	–	F/F	r/r MM	148	Higher RR Longer PFS (in multivariate Cox regression analysis)	van de Donk et al. (18)
Trastuzumab	H/H	V/V	mBC	54	Higher RR Longer PFS	Musolino et al. (19)
Trastuzumab	–	V carriers	early-stage BC	1156	Longer DFS	Gavin et al. (20)
Trastuzumab	H/H	–	early-stage/mBC	15/35	Higher RR Longer PFS (only in mBC group)	Tamura et al. (21)
Trastuzumab	H/H or H/R	–	BC	132	Higher EFS	Roca et al. (22)
Trastuzumab	H/H	V/V	GC	42	Longer PFS (for H/H genotype) Higher DCR (for the combination of H/H and V/V genotype)	Wang et al. (23)
Margetuximab	–	F carrriers	mBC	506	Longer PFS	Rugo et al. (24)
Cetuximab	H/H	V/V	mCRC	69	Longer PFS	Bibeau et al. (25)
Cetuximab	–	V carriers	mCRC	52	Higher RR Longer OS	Etienne-Grimaldi et al. (26)
Cetuximab	H/H or H/R	V carriers	mCRC	96	Higher RRLonger PFS (for V/- allele)	Trotta et al. (27)
Cetuximab	H/H	F/F	mCRC	595	Longer OS(for H/H genotype only or in combination with F/F genotype)	Shepshelovich et al. (28)
Cetuximab	H/H	–	mCRC	293	Longer PFS and OS	Liu et al. (29)
Cetuximab	H/H or H/R	F carriers	mCRC	39	Longer PFS	Zhang et al. (30)
Cetuximab	–	F carriers	advanced CRC	58	Longer OS	Dahan et al. (31)
Cetuximab	H/H	–	mCRC	106	Higher DCR	Rodriguez et al. (32)
Cetuximab	H/H	V/V	recurrent and mHSNCC	103	Longer PFS and OS	Magnes et al. (33)
Farletuzumab	H/H	V/V	EOC	461	Longer PFS in patients with low baseline CA125 levels	Wang et al. (34)
Ipilimumab	–	V carriers	Melanoma	121	Higher RR	Van Allen et al. (35) Snyder et al. (36)
Ipilimumab	–	V carriers	Melanoma	51	Longer OS	Snyder et al. (36)

mAb, monoclonal antibody; mBC, metastatic breast cancer; EOC, epithelial ovarian cancer; mCRC, metastatic colorectal cancer; DLBCL, diffuse large B cell lymphoma; FL, follicular lymphoma; mHSNCC, metastatic head and neck squamous cell carcinoma; MCL, mantle cell lymphoma; r/r MM: relapsed/refractory multiple myeloma; NHL, non-Hodgkin lymphoma; EFS, event-free survival; DCR, disease control rate; OS, overall survival; D/PFS, disease/progression free survival; RR, response rate.

## FC Gamma Receptor Function and Polymorphism

FcγRs are members of the Ig family and are expressed mainly by hematopoietic cells. They bind to the Fc portion of IgG and are essential in the control of both humoral and innate immune responses (37). These receptors may be classified by their affinity for IgG and signalling activities [reviewed by (38)]. There are two functionally defined subtypes of human FcγRs: activating and inhibitory. Activating FcγRs include FcγRI (CD64), FcγRIIa (CD32a), FcγRIIc (CD32c), FcγRIIIa (CD16a), and FcγRIIIb (CD16b). Some activating human FcRs, such as FcγRIIa and FcγRIIc, carry their own immunoreceptor tyrosine-based activating motif (ITAM) in their intracytoplasmic domains to initiate cellular activation. FcγRIa and FcγRIIIa are associated with signaling adaptor FcR-γ chain or CD3zeta chain that contain ITAM for signaling transduction while FcγRIIIb is a glycophosphadylinositol-linked surface receptor that requires the associated MAC-1 (CD11b/CD18) for activation signal transduction [reviewed by (9)]. Inhibitory FcγRIIb (CD32b) possess an immunoreceptor tyrosine-based inhibitory motif in their intracytoplasmic domain, which serves to limit responses through the activation of FcγRs as well as other stimulatory receptors [reviewed by (39)]. Preclinical studies have established that the ratio of activating to inhibitory receptor engagement determines FcγR-dependent antibody-mediated target cell depletion (40).The magnitude of the activation or inhibition of the immunological response triggered by FcγRs not only depends on the corresponding signalling pathways, but also on the level of expression and the affinity for the ligand. High-affinity FcγRI is constitutively expressed by monocytes and macrophages, and its expression can be induced on other myeloid cells under inflammatory conditions. Its binding produces ADCC and it is able to bind to immune complexes or free/monomeric Ig, regulating plasma IgG levels (41). FcγRII and FcγRIII are low affinity receptors that can only bind to aggregated or opsonized IgG and immune complexes, and can trigger a range of effector and immunoregulatory functions, including degranulation, phagocytosis and regulation of antibody production. FcγRIIa is widely expressed on all subsets of the myeloid lineage but is absent from lymphocytes. It has an important role in phagocytosis of IgG2-opsonized targets. FcγRIIb is predominantly expressed in B-lymphocytes, and is involved in inhibiting signalling from the B-cell receptor (BCR). There is also evidence that FcγRIIb is not the only FcγR capable of mediating inhibitory signalling, with inhibitory ITAM signalling being observed downstream of activatory FcγR ligation in some circumstances [reviewed by (42)]. FcγRIIc mRNA and protein have been detected in B cells from healthy FcγRIIc-57Q donors, which counterbalances the negative feedback of FcγRIIb and thus regulated cell’s activation threshold (43). NK cells, monocytes and neutrophils may also expressed FcγRIIc although its functionality is not yet fully determined [reviewed by (38)]. FcγRIIIa induces NK cell-mediated ADCC and is also responsible for the clearance of immune complexes by macrophages. Activating FcγRIIIa on macrophages are important for mediating ADCP. *In vitro* studies of tratuzumab-mediated ADCP of HER2-overexpressing tumor cells demonstrate that FcγRIIIa has greater influence than FcγRIIa (7). FcγRIIIb is selectively present on neutrophils and on a subset of basophils and plays a major role in the secretion of toxic products in response to immune complexes (44). Available preclinical evidence suggests that FcγRIIIb cooperates with activating FcγR, like FcγRIIa/c, to promote phagocytosis of opsonized microbes (44). However, in the context of cancer FcγRIIIb likely serves as a decoy receptor for IgG, likewise competing with FcγRIIa for the binding of therapeutic antibodies, thereby resulting in decreased ADCC (45). The different characterization of human FcγRs and their known functional effects are shown in **Table 2**.

**Table 2:** Overview of FcγRs and their functional effects (Modified from Bournazos et al. and Bruhns et al.).

IgG receptor	FcγRI	FcγRIIa	FcγRIIb	FcγRIIc	FcγRIIIa	FcγRIIIb
**Affinity for IgG (Ka)**	High (10^9^-10^10^/M)	Low (<10^7^/M)	Low (<10^7^/M)	Low (<10^7^/M)	Low (<10^7^/M)	Low (<10^7^/M)
**Specificity for IgG subclass**	IgG1 = IgG3 >>> IgG4	IgG1 >>> IgG3 > IgG2 > IgG4	IgG3 = IgG4 > IgG1 > IgG2 (only I232 for polymorphism)	IgG3 = IgG4 > IgG1 > IgG2 (only Q13 for polymorphism)	IgG3 >>> IgG1 = IgG4 > IgG2	IgG3 > IgG1
**Cellular expression**	Monocytes, macrophages, subset of dendritic cells and PMN (IFN-γ or G-CSF-induced)	PMN, monocytes, macrophages, platelets and subset of endothelial cells	Monocytes, macrophages, dendritic cells, basophil and B cells	Monocytes, macrophages, NK cells	Monocytes, macrophages, NK cells and gamma-delta T-cells	Neutrophils and basophils
**Relevant SNP**	c. -131C>G; c.845-23_845-17delTCTTTG; D324N	R131H	I232T	Q57X	V158F	HNA-1a
**Signalling activities**	Activation	Activation	Inhibition	Activation	Activation	Activation
**Functional effect**	Regulation of IgG levels, phagocytosis and ADCC	Induces phagocytosis and mediator release	Regulation of B cells and phagocytosis	Mechanism unknown	Phagocytosis and ADCC	Phagocytosis
**Functional effect of polymorphism**	-131G: higher promoter activity; c.845-23_845-17delTCTTTG: lower CD64 expression; N324: higher degranulation and more pro-inflammatory cytokine production	H131: higher affinity for IgG2	T232: decreased inhibitory activity	Truncated non-functional protein	V158: higher affinity for IgG1 and IgG3, bind IgG4	NA1: higher degranulation and more phagocytosis

IFN-γ, interferon-γ; G-CSF, granulocyte colony-stimulating factor; NK cells, NK cells; PMN, polymorphonuclear leucocytes; SNP, single nucleotide polymorphism.

The different isotypes of IgG have different affinities for FcRs, conditioning their biological effect. IgG2 and IgG4 interact poorly with FcγRs, whereas IgG1 and IgG3 show a higher affinity (**Table 2**). In addition, allelic variants of FcγRs located in extracellular Ig-like domains that bind to IgG, can affect the binding affinity between the FcγR and IgG subclass. FcγRIIa contains a single nucleotide polymorphism (c.519G>A) at codon 131 that results in an arginine (R) to histidine (H) substitution in the second Ig-like domain (46). Previous studies have reported that FcγRIIa-131H allele has a higher binding affinity for IgG3 and IgG2 than FcγRIIa-131R (47). Sanders et al. reported a higher capacity for IgG2-mediated phagocytosis in neutrophils carrying the 131H/H variant compared with those carrying the 131R/R genotype (48). Moreover, the FcγRIIa-131H allele is involved in susceptibility to autoimmune disease [reviewed by (49)]. The substitution of phenylalanine (F) to valine (V) at position 158 (c.559T>G) in extracellular domain 2 of FcγRIIIa originates a variant (158V) with a higher affinity for IgG1 and IgG3 than the 158F variant, and permits IgG4 binding (50). Wu et al. showed that IgG-stimulated NK cells from individuals with FcγRIIIa homozygous for the V allele elicited a larger flux in intracellular calcium, a greater degree of cell activation, and a more pronounced program of activation-induced cell death than those with the F/F genotype (51). Recently, Nyborg et al. demonstrated that the FcγRIIIa polymorphic 158V variant has an approximately 10-fold higher affinity for IgG1 than FcγRIIIa-158F (52). For both SNPs referred above, it was also reported that the higher binding affinity correlated with enhanced lytic activity of effector cells and increased cytokine release in several preclinical studies (51, 53, 54). In leukocyte–based *in vitro* assays, Hussain et al. reported a stronger IFN-γ response to mAbs in donors homozygous for both FcγRIIa-131H and FcγRIIIa-158V alleles compared to donors homozygous for the low affinity alleles (54). Other less prevalent SNPs in Caucasian populations (<2%), such as the homozygous FcγRIIb-232-T genotype (c.695T>C), appear to affect inhibitory BCR signalling (55). Several reports have shown that FcγRIIb-232-T/T is associated with susceptibility to autoimmunity (56). Recently, three novel variants within the FcγRIa gene have been reported (c.-131C>G, c.970G>A or FcγRIa-p.D324N, c.845-23_845-17delTCTTTG). Genetic analyses revealed that FcγRIa genotypes were significantly associated with sarcoidosis susceptibility and severity (57). **Table 2** shows the most relevant FcγR polymorphisms and their functional effects [reviewed by (38)]. These polymorphic variants can significantly influence the effectiveness of both ADCC and immunotherapeutic regimens containing mAbs [reviewed by (58)].

On the other hand, several studies have demonstrated that genes coding for FcγRIIc, FcγRIIIa and FcγRIIIb exhibit copy number variations (CNV) (38). These CNV can alter the level of FcγR expressed at the cell surface and thus affect cellular function through gene dose mechanisms [reviewed by (10)]. For FcγRIIc, van der Heijden et al. have demonstrated an association between CNV and surface expression of FcγRIIc-ORF (FcγRIIc-open reading frame) allele in NK cells, leading to the activation of NK cell-mediated ADCC (59). CNVs of FcγRIIIb are very common in human populations. Numerous studies show that FcγRIIIb CNV are significantly associated with a number of inflammatory diseases and it role in immune responses is well established (60, 61). These studies reported a link between FcγR CNV and disease pathogenesis, and so it of interest to determine whether these genetic rearrangement have an impact on disease such as autoimmunity, cancer or infection.

## Rituximab

Rituximab is a chimeric IgG1 mAb directed against CD20, an antigen expressed by B cells (62). It induces B-lymphocyte depletion through at least four pathways: ADCC, ADCP, complement-dependent cytotoxicity (CDC), and direct antitumor effects *via* either apoptosis or other cell death pathways (63). Rituximab maintenance, either after induction with rituximab alone, or with chemotherapy, has demonstrated a clinical benefit in patients with hematological diseases [reviewed by (64)]. Although its efficacy for the treatment of B-cell hematologic malignancies is well established, some patients do not respond to first-line treatment, and others experience relapse after initial response to therapy (64). Several factors have been suggested to explain failures (65-67), including FcγR genetic polymorphisms and FcγR protein expression level (11–14). For example, it has been well-documented FcγRIIb promoting resistance role to antibody therapy when expressed on tumor B cells (68, 69). FcγRIIb-mediated internalization of anti-CD20 reduces phagocytosis of tumor B cells (68) and, thus, high FcγRIIb expression can be associated with shorter survival in patients with B-cells malignances after rituximab-containing regimens (70).

*In vitro* studies suggest that FcγR polymorphisms are associated with response to rituximab. A study conducted by Dall´Ozzo et al. demonstrated that FcγRIIIa-158V genotype displays a higher affinity for rituximab than FcγRIIIa-158F genotype by comparing rituximab concentrations inhibiting the binding of anti-CD16 with V/V NK cells and NK cells homozygous for FcγRIIIa-158F/F (71). When the efficacy of B-cell lysis by NK cells obtained from healthy donors is analysed, the rituximab concentration resulting in 50% lysis (EC50) observed with NK cells from V/V donors was 4.2 times lower than that observed with NK cells from F/F donors (71). Based on the observed influence of FcγRIIIa polymorphisms *in vitro*, several retrospective studies in patients with non-hodgkin lymphomas (NHL) treated with rituximab have looked for a possible correlation between FcγRIIIa polymorphisms and clinical outcomes (11–14).

Carton et al. reported the results of a study including 49 patients with untreated follicular lymphoma (FL) (11, 12). They found that FcγRIIIa-158V/V patients (20% of population) had an improved response rate (RR) at 2 months and 12 months compared with FcγRIIIa-158F carriers (100% vs 67% and 90% vs 51%, respectively; p=0.03). However, there was a non-statistically significant difference in progression free survival (PFS) (11). Weng et al., in a study including 87 patients with FL, reported that patients with the FcγRIIIa-158V/V genotype showed higher RR to rituximab compared with 158V/F and 158F/F genotypes at 6, 9 and 12 months (85% vs 45%, 75% vs 36% and 75% vs 26%, respectively; p<0.05) (12). They also found that patients with FcγRIIa-131H/H showed a significantly higher RR than carriers of the 131R allele at 6, 9, and 12 months (80% vs 43%, 70% vs 32% and 55% vs 26%, respectively; p<0.05). In addition, a statistically significant improvement in PFS at 2 years was observed for patients with FcγRIIIa-158V/V and FcγRIIa-131H/H genotype (45% for V/V vs 14% for F carriers and 37% for H/H and 14% for R carriers; p<0.05) (12). Ghielmini et al. reported that the FcγRIIIa-158V/V genotype constituted an independent factor for higher event-free survival (EFS), but not RR, in patients with FL and mantle cell lymphoma (MCL) treated with single-agent rituximab (13).

The SWOG trial evaluated 66 FL patients treated with anti-CD20 mAb-containing regimens [rituximab (n=26) or tositumomab (n=40)] and evidenced an improved overall survival (OS) in carriers of the FcγRIIIa-158V allele compared with those with the F/F variant (Hazard ratio (HR)=0.33, 95% CI, 0.11-0.96, p=0.042), with 5-year OS of 100%, 97% and 75% for V/V, V/F and F/F, respectively. There were no differences with regards to the FcγRIIa polymorphisms (14). Kim et al. observed, in 113 patients with diffuse large B-cell lymphoma (DLCBL) receiving standard therapy, that significantly higher complete (CR) and overall response rates (ORR) were associated with the FcγRIIIa-158V/V genotype, compared with FcγRIIIa-158V/F or F/F (CR: 88% in V/V vs 79% in V/F vs 50% in F/F; ORR: 98% in V/V vs 90% in V/F vs 50% in F/F; p=0.002) (15). Zhang et al. confirmed these data in 34 patients with DLCBL, with a 1-year OS in the group with FcγRIIIa-158V/V or V/F genotype higher than group with F/F genotype (80% in V/V and V/F vs 60% in F/F p<0.05) (16). However, in both studies, patients clinical outcome was not affected by FcγRIIa genotypes.

A meta-analysis of 10 studies involving 1050 patients of European and Asian origin (472 patients with DLBCL and 578 patients with another NHL), investigated a possible association between the FcγRIIIa polymorphism and non-responsiveness to rituximab-based therapy in NHL (17). The data showed that Asian patients with the FcγRIIIa-158V allele have a significantly higher CR rate compared with those with the F/F genotype (p<0.05), confirming an association between the FcγRIIIa-158F/F genotype and a poor response to rituximab-based chemotherapy in NHL Asian patients (17).

PRIMA and RESORT trials evaluated the influence of FcγRIIIa and FcγRIIa polymorphisms in FL patients treated with rituximab. The PRIMA study included 460 previously untreated patients with high tumour burden that received the usual induction immunochemotherapy and maintenance with rituximab, whereas in the RESORT trial, 408 previously untreated patients with low tumour burden were treated with single-agent rituximab. Both trials reported that FcγRIIIa and FcγRIIa polymorphisms did not influence therapeutic outcomes of rituximab in patients with FL, either combined with chemotherapy or used as maintenance treatment (72, 73). Some previous small retrospective studies of rituximab in FL were also consistent with these results (74–76). Concerning DLBCL, Ghesquiéres et al. evaluated the prognostic value of FcγRIIIa and FcγRIIa in two prospective cohorts from LYSA (n=554) and SPORE (n=580) trials of patients treated with anthracycline–based chemotherapy and rituximab (77). They found no association between FcγRIIIa and FcγRIIa SNPs and EFS or OS. Recently, in patients with previously untreated advanced FL or DLBCL, these results have been confirmed in the GALLIUM (n=1202) and GOYA (n=1418) trials that assessed the potential impact of these FcγR genotypes on the efficacy of obinutuzumab or rituximab in combination with chemotherapy, respectively (78). Several previous smaller studies agree with these results (79–84). According to the authors, one explanation for the lack of prognostic value for FcγRIIIa genotypes in the context of immunochemotherapy is that the association between chemotherapy and rituximab is deleterious for ADCC effectors (77). On the other hand, in a meta-analysis using an ordinal model, these authors reported that the FcγR–IIa-131R allele was associated with a better EFS (HR=0.87; 95%CI, 0.76–0.99; p=0.04) and OS (HR=0.86; 95%CI, 0.73–1.00; p=0.05). One hypothesis for this unexpected outcome with the low affinity FcγRIIa-131R/R could be that this SNP causes a reduced clearance of rituximab that increases anti-CD20 availability (77).

In patients treated with rituximab-containing regimens for chronic lymphocytic leukaemia (CLL) there is no evidence of an association between FcγR polymorphisms and clinical outcomes (85, 86).

In conclusion, the prognostic effect of FcγRIIIa has mainly been observed in FL patients treated with single agent rituximab (11–13) or by immunochemotherapy (14) in small retrospective studies. Nonetheless, prospective clinical trials have not identified a clear prognostic impact for FcγR polymorphisms in patients treated with anti-CD20 mAb (72, 73, 77, 78). Discrepancies between the different studies can be attributed to many factors, including biologic heterogeneity, clonal evolution, tumour bulk, prior treatments, numbers of patients and host factors. It is also important to note that CNVs of FcγRIIIa were not determined in these studies, which could significantly affect the homozygous counts of FcγRIIIa genotypes. In addition, the existence of other factors or mechanisms of resistance may influence the efficiency of ADCC in tumour eradication (87).

Thus, the use of FcγR polymorphisms as predictive biomarkers of response to rituximab is still inconclusive in hematological disorders.

## Daratumumab

CD38 is a type II transmembrane glycoprotein highly expressed in multiple myeloma (MM). Daratumumab is a CD38-targeting IgG1 antibody. The efficacy of daratumumab both as monotherapy and in combination with standard-of-care regimens in MM has been established in clinical trials (18).The mechanisms of action by which daratumumab exerts its antitumor effects include tumour cell apoptosis upon FcγR cross-linking, CDC, ADCC and ADCP (88).

In the GEN501 and SIRIUS studies, 148 relapsed/refractory (r/r) MM patients received daratumumab as single agent. Analysis of FcγRIIa and FcγRIIIa polymorphisms were performed in 96 and 94 patients, respectively. An increased RR was observed among patients treated with daratumumab with the FcγRIIIa-158F/F polymorphism (≥ partial response (PR): 47.6%), versus those with the FcγRIIIa-158V/F (≥PR: 20.0%) and V/V polymorphisms (≥PR: 20.0%) (p=0.0049). In multivariate Cox regression analysis, the FcγRIIIa-158F/F polymorphism was significantly associated with improved PFS [HR=1.65 (95% CI, 1.04-2.61), p=0.033]. The FCGR2A-131H/R polymorphism did not significantly predict clinical outcome to daratumumab therapy. In both univariate and multivariate analysis, OS was not affected by the Fc receptor genotype subsets. They concluded that selecting patients based on FcγR genotype alone does not seem to be warranted, given that patients with less favourable genotypes may also derive marked clinical benefit from daratumumab treatment (89). It is important to highlight that daratumumab treatment leads to rapid depletion (85%) of CD38^pos^ NK cells, lasting up to 6 months following cessation of treatment (90). Therefore, ADCC may play a lesser biological role than originally thought in terms of clinical response to daratumumab.

## Trastuzumab

HER2, also known as Neu, ErbB-2, CD340 or p185, is an oncoprotein amplified or over-expressed in ~30% of breast cancers (BC) and other tumours, strongly associated with increased disease recurrence and worse prognosis (91). Ligand binding can induce homo- and heterodimerization among the different ErbB family receptors, inducing a downstream signal that leads to cell transformation and cancer. Trastuzumab is a fully humanized IgG1 mAb targeting the extracellular domain of the HER2 that has demonstrated clinical efficacy in both early and advanced BC (92, 93), metastatic gastric cancer (GC) (94), and urothelial cancer (UC) (95) with overexpressed HER2. The success of trastuzumab is attributed to multiple mechanisms that involve antibody binding to HER2 on the cancer cell membrane [reviewed by ref (96): it prevents homo- and heterodimerization, thus inhibiting the mitogen-activated protein kinase (MAPK) and phosphatidylinositol 3-kinase (PI3K/Akt) pathways. This blocks the cell cycle and suppresses cell growth and proliferation (97); it inhibits proteolytic cleavage of the extracellular receptor domain (98); it disrupts interaction between HER2 and SRC tyrosine kinase, inhibiting PTEN activation-mediated cell proliferation (99); it acts as an anti-angiogenic (100); it kills trastuzumab-bound tumour cells by ADCC activation, followed by internalization and degradation of HER2 (101). Moreover, ADCC also activates tumour-antigen-specific cellular immunity *via* intercellular crosstalk among NK and dendritic cells, which may also enhance the efficacy of tratuzumab therapy (102). However, not all HER2+ BC patients respond to trastuzumab as primary or acquired resistance to trastuzumab can appear before the end of the first year of treatment in a metastatic setting (92, 93).

Menyhart et al. reported HER2 amplification, impaired access to the binding site, augmented signalling through other ErbB family receptors and their ligands, activation of HER2 targets by alternate heterodimers, signalling triggered by downstream members, altered expression of cell cycle and apoptotic regulators, hormone receptor status, resistance to ADCC (FcγR polymorphism), and altered miRNA expression signatures as possible trastuzumab resistance biomarkers in BC (103). In a clinical setting, the influence of the FcγR polymorphism on ADCC activity as a biomarker of trastuzumab response has been studied.

A pre-clinical study showed that peripheral blood mononuclear cells (PBMCs) with FcγRIIIa-158V/V and/or FcγRIIa-131H/H genotypes exert significantly higher trastuzumab-mediated cytotoxicity than PBMCs with other genotypes (19). Shimizu et al. evaluated the effect of the FcγR genotype on immune-related gene expression of PBMCs in patients with HER2+ metastatic BC treated with single-agent trastuzumab. One week after starting trastuzumab, thirty gene sets related with an early systemic response mediated by macrophages and NK cells were identified in the cohort of FcγRIIIa-158V/V variants, whereas no gene set was identified in the cohort of non-V/V variants. At week eight, eleven different gene sets, associated with an immune reaction involving other components of PBMCs such as neutrophils and eosinophils were enriched for FcγRIIa-131H/H, but none in non-H/H variants. In contrast, no immune-related gene sets were observed at baseline (104). These results indicate that FcγR polymorphisms could contribute to systemic immune reaction triggered by trastuzumab.

Musolino et al. reported the beneficial effect of FcγRIIIa-158V/V in a study of 54 patients with HER-2/neu-amplified metastatic BC treated with trastuzumab plus taxane (19). A significant difference in ORR and PFS was observed between patients with FcγRIIIa-158V/V and patients with either the 158V/F or 158F/F genotype (ORR: 82% vs 42%, vs 35%, respectively; p=0.03, and PFS: Not reached vs 15.0, vs 11.1 months, respectively; p<0.05). Moreover, a multivariate analysis demonstrated that the combination of the two favourable genotypes (V/V and/or H/H) was independently associated with better ORR (71% vs 38%; p=0.04) and PFS (30.3 vs 12.8 months; p=0.01) (19). Gavin et al. reported a large cohort of patients with HER2+ early-stage BC (n=1156), enrolled in the NSABP B-31 phase III clinical trial, that received doxorubicin and cyclophosphamide followed by paclitaxel and trastuzumab (20). Patients who had the FcγRIIIa-158V/V or V/F genotype received a greater benefit from trastuzumab (HR=0.31; 95%CI, 0.22-0.43; p<0.001) than patients homozygous for the low-affinity allele (HR=0.71; 95%CI, 0.51-1.01; p=0.05) (20). The authors concluded that patients homozygous for FcγRIIIa-158F benefited less from the addition of trastuzumab to chemotherapy than V/V patients, suggesting that ADCC plays a role in determining the efficacy of trastuzumab in HER2+ BC adjuvant treatment.

Tamura et al. evaluated the association between FcγR polymorphisms and clinical outcomes in a cohort of 15 operable and 35 metastatic HER2+ BC patients treated with chemotherapy and trastuzumab in a prospective study (21). In both neoadyuvant and metastatic settings, the pathological response and ORR were significantly higher in patients with FcγRIIa-131H/H genotype than in those with 131H/R or 131R/R (71% for H/H vs 0% for H/R + R/R, p=0.015; 40% for H/H vs 12% for H/R + R/R, p=0.043, respectively). Patients with H/H presented a significantly longer PFS than those with R/- in a metastatic setting (9.2 vs 3.5 months, p=0.034) (21).

Roca et al., in a prospective clinical trial performed in 132 patients with HER2+ BC and treated sequentially with adjuvant chemotherapy and trastuzumab (UNICANCER-PACS04), observed that the presence of the H allele was associated with a better EFS than in R/R genotype patients at 5 years of follow-up (90.3% vs 69.7%; p=0.027) (22).

In contrast to the findings of these relatively small studies, Hurvitz et al. and Norton et al. found no correlation between FcγRIIa-131H/R and FcγRIIIa-158V/F SNPs and clinical outcome in a large retrospective cohort of over 1000 early HER2+ BC patients treated with adyuvant trastuzumab (105, 106). Nevertheless, in Norton et al. study was found that FcγRIIb-232I/I patients showed superior DFS in the trastuzumab arms as compared to patients treated with chemotherapy alone (p<0.0001) (106). In the previously cited paper, Gavin et al. attributed the observed discrepancies to: 1) distinct regimen, timing of treatment and differences in patient selection criteria (in the Norton et al. study); 2) sampling bias in both the previous FcγR studies; 3) failure of the FcγRIIIa heterozygotes to satisfy the Hardy-Weinberg equilibrium (HWE) in the study of Hurvitz et al. (20). If a selected genotype distribution in the population misses the HWE, the results should be treated cautiously because the observed genotype distribution does not represent that of a healthy population (103).This lack of fulfilment of the HWE law in the investigated population might be influenced by additional factors, such as an incorrect assumption of the model, non-random mating, or a sampling error: small sample size or population not well defined (diverse ethnic groups) (107). All these biases can mask a possible correlation between FcγR polymorphisms and clinical outcomes.

At this time, evidence for an association between FcγR genotype and clinical benefit of HER2+ BC patients that receive trastuzumab-based therapy is still inconclusive and further clinical trials are required in this setting.

Wang et al. examined 42 HER2+ GC patients receiving chemotherapy and trastuzumab (23). They found a significantly higher PFS in patients with FcγRIIa-131H/H genotype compared to the H/R or R/R genotype (p=0.001). When combining FcγRIIa and IIIa polymorphisms, they found that H/H or V/V genotypes were associated with a significantly improved disease control rate (95.2% vs 71.4%, p=0.04) and PFS (p<0.001) compared to other genotypes. They concluded that FcγR polymorphisms might predict clinical outcome in metastatic GC patients receiving trastuzumab treatment. Nonetheless, these findings must be verified by larger studies and evaluated in Caucasian patients (23).

## Cetuximab

EGFR is a member of the ErbB tyrosine kinase family that modulates cell proliferation, survival, adhesion, migration and differentiation. EGFR forms a homodimer or heterodimer with other members of the Erb family (ErbB2, ErbB3 and ErbB4) and activates the downstream signalling through the MAPK cascade and the PI3K/Akt/mTOR pathway (108). EGFR is abnormally upregulated or activated in a variety of tumours (109). In recent years, the use of anti-EGFR mAbs has played an increasing role in the treatment of several solid tumour types including non-small-cell lung carcinoma (NSCLC), colorectal cancer (CRC), and head and neck squamous cell carcinoma (HNSCC) in which KRAS mutation is not detected. Besides inhibiting the EGFR pathway, anti-EGFR mAbs may exert anti-tumour effects through ADCC (110). This is the case of cetuximab, a chimeric IgG1 mAb directed to the extracellular domain of the EGFR, which has been approved to treat KRAS wild-type metastatic CRC (mCRC) (111) and HNSCC (112).

Although cetuximab has shown promising efficacy in mCRC patients, the molecular mechanisms underlying clinical activity or resistance to cetuximab are not well known to date. FcγRIIa and FcγRIIIa polymorphisms have been investigated as molecular markers that predict cetuximab response, OS, and toxicity in mCRC patients. In a retrospective study, the association between FcγR polymorphisms and KRAS mutation with outcome in 69 irinotecan-refractory mCRC patients treated with cetuximab plus irinotecan was examined (25). Longer PFS were observed in patients with FcγRIIa-131H/H and/or FcγRIIIa-158V/V genotypes compared to 131R and 158F carriers (5.5 v 3.0 months; p=0.005). Moreover, KRAS mutation and FcγR combined status were independent variables for PFS (25). Etienne-Grimaldi et al. (26), in a prospective study, performed a multifactorial pharmacogenetic analysis, including FcγRIIa and FcγRIIIa polymorphisms, in 52 patients receiving cetuximab in combination with irinotecan and tegafur-uracil (UFT) plus folinic acid. These authors reported that patients carrying the FcγRIIIa-158V allele exhibited a higher RR (62.1% response in F/V or V/V vs 26.3% in F/F, p=0.02) and OS (20.9 months in F/V or V/V vs 12.4 months in F/F, p=0.032), respectively (26).

Trotta et al. analyzed 96 consecutive patients with mCRC at diagnosis in a study that assessed FcγR status and *in vitro* cetuximab-mediated ADCC (27). Patients carrying the FcγRIIa-131H allele (H/H and H/R) and FcγRIIIa-158V allele (V/V and V/F) displayed higher ADCC *in vitro* compared to patients carrying the 131R/R (p=0.013) and the 158F/F genotype (p=0.001), respectively. Moreover, PFS of patients with an FcγRIIIa-158V allele was significantly longer compared to patients carrying 158F/F (p=0.05), whereas no significant difference was observed for OS. These authors suggest that the *in vitro* evaluation of basal NK activity may help to predict the therapeutic response of mCRC patients (27).

More recently, a retrospective study (CCTG CO.20) performed in 595 patients with metastatic wild-type KRAS CRC treated with cetuximab found an improved OS in the FcγRIIa-131H/H genotype group (n=165) compared to those with R/‐ genotype (n=427) (HR=0.66; p<0.001; median absolute benefit, 1.3 months) (28). These results replicate those previously described in the CCTG CO.17 randomized controlled trial (29). These authors concluded that FcγRIIa is a promising biomarker for clinical management in these patients.

These data are in discrepancy with the results previously reported by Zhang et al. and Dahan et al., which demonstrated that advanced CRC patients with FcγRIIIa-158V/V genotype presented a dramatically shorter OS (in the entire population and in wild-type KRAS patients) (30, 31). More recently, in a comprehensive meta-analysis containing 2831 patients with mCRC treated with anit-EGFR mAbs, Ying et al. showed that the F/F genotype was significantly associated with a longer PFS than VF/VV genotype (median survival ratio (MSR)=0.680, 95%CI=0.549-0.842 in overall population; MSR=0.728, 95%CI=0.648-0.818 in wild-type KRAS population), and a longer OS than those carrying the V/V genotype (MSR=0.733, 95%CI=0.578-0.930 in overall population) (113). These differences with the previously described results (25–27) could be due to the relatively limited sample size of these studies or cetuximab treatment regimen, among other causes. For example, cetuximab was given as third-line monotherapy in the Zhang study contrary to Etienne-Grimaldi report where the patients received cetuximab combined with irinotecan and UFT plus leucovorin as first-line.

In summary, the data support the use of FcγRIIa polymorphism to predict the response to cetuximab in mCRC, whereas FcγRIIIa polymorphism studies are still controversial. All studies agree that the H/H variant is the most beneficial for FcγRIIa genotype (25, 27, 28, 30, 32), and two report evidence that patients with the H/R genotype have a better clinical outcome (27) (30). Moreover, the role of the FcγRIIIa polymorphism in modifying the primary relationship between FcγRIIa and clinical outcomes is unknown. Two studies have shown that the combination of FcγRIIa-131H/H and FcγRIIIa-158F/F genotype can improve clinical outcomes (28, 30). Differences in patients’ characteristics, study design, therapeutic protocols, distribution of genotypes in the different patient groups, and even methodological problems (deviation from the HWE) (25, 114) might, in part, explain the discrepancies between these studies.

Some data show beneficial cytotoxic effects of effector cells with FcγRIIIa-158V alleles in HNSCC cell lines (115). In a clinical setting, the influence of FcγRIIa and FcγRIIIa polymorphisms on the survival of recurrent or metastatic HNSCC patients has been evaluated in a cohort of 103 patients treated with cetuximab, cisplatin or carboplatin and fluorouracil as palliative first-line chemotherapy (33). Magnes et al. observed that survival of patients with FcγRIIa-131H/H and/or FcγRIIIa-158V/V genotypes improved significantly compared to patients carrying 131R and 158F alleles (median PFS: 5.5 vs 4.1 months, p=0.02; median OS: 10.2 vs 7.2 months, p=0.04). This advantage is maintained in the subset of patients with p16-positive tumours (median PFS 10.3 months for patients with 131H/H and/or 158V/V genotypes vs 4.0 months for 131R and 158F carriers, p=0.02) (33), but has not been confirmed in other series (116, 117).

With current findings, FcγR polymorphisms, especially FcγRIIa-131H/H, can be a useful tool for selecting candidates for treatment with anti-EGFR inhibitors in mCRC and HNSCC.

## Farletuzumab

Farletuzumab is a humanized IgG1 mAb that targets human folate receptor-α (FRα), which is overexpressed in most epithelial ovarian cancers (EOC) but is largely absent from normal tissue (118). A preclinical study has demonstrated that the anti-tumour effect of anti-FRα mAbs against an experimental model of EOC is mediated by its ADCC activity, since anti-tumour effect is completely abolished when FcR binding domain is modified (119). Recently, a randomized, double-blind, placebo-controlled phase III study (MORAb-003-004) with 1100 patients investigated the effect of standard therapy (carboplatin plus either paclitaxel or docetaxel) with either farletuzumab (1.25 mg/kg or 2.5 mg/kg) or placebo on PFS in EOC patients in a platinum-sensitive first relapse (120). Prespecified subgroup analyses demonstrated that patients with CA125 levels no more than three times the upper limit of normal (ULN) and patients with higher farletuzumab exposure (2.5 mg/kg) showed superior PFS and OS compared to placebo (HR=0.49; p=0.0028; HR=0.44; p=0.0108, respectively) (120). Wang W. et al. performed a *post hoc* analysis of FcγRIIa-131 and FcγRIIIa-158 codon in 461 patients (MORAb-003-004 trial) (34), reporting that farletuzumab showed enhanced binding to FcγRIIIa-158V high-affinity receptor. In addition, an increase in PFS was observed in patients with low baseline CA125 levels and at least one high-affinity allele of FcγRIIa-131 or FcγRIIIa-158 (34).

The findings suggest that farletuzumab clinical outcome depends on CA125 level, while the FcγRIIa and FcγRIIIa high- versus low-affinity receptor types may also contribute to farletuzumab-mediated clinical outcome.

## Immune Checkpoint Inhibitors

Immune checkpoints are molecules that control the immune system to maintain self-tolerance and modulate the duration and amplitude of physiological immune response in peripheral tissues, in order to minimize collateral tissue damage (121). CTLA-4 and PD-1 are immune checkpoints expressed on the surface of activated T cells. Moreover, CTLA-4 is also constitutively expressed in high levels in CD4+CD25+ T regulatory (Treg) cells (122). CTLA-4 is a type I transmembrane glycoprotein that presents homology to CD28 and down-regulates T-cell activation (122). The PD-1 receptor is a negative regulator of T cell activity that binds with two ligands, PD-L1 and PD-L2, and is involved in the control of T cell immune responses (123). PD-L1 and PD-L2 are expressed in antigen presenting cells and may be expressed by tumours or other cells in the tumour microenvironment. Activation of the PD-1/PD-L1 axis contributes to suppression of anti-tumour immunity and serves as a mechanism for tumour evasion (124). For this reason, several mAbs that block these inhibitory pathways have been developed in recent years, with the aim of enhancing immune system activity, as immunotherapy against different tumors (124). Ipilimumab, a CTLA-4 blockade mAbs, PD-1 inhibitors nivolumab and pembrolizumab, as well as PD-L1 inhibitors atezolizumab, durvalumab and avelumab have been approved by the FDA for treatment of a wide variety of solid and hematologic tumours [see (2) review].

Recent findings highlight the importance of Fc-FcγR interactions to carry out ICIs mAb activity *in vivo*. Dahan et al. reported that anti-PD-1/PD-L1 mAbs differ in their FcγR requirements for their *in vivo* activity. This preclinical study showed that some Fc-FcγR interactions (IgG2a and IgG1) can be detrimental to the therapeutic efficacy of anti-PD-1 mAbs, by facilitating macrophages to deplete PD1+ effector T cells. Optimal anti-tumour activity of mAbs targeting PD-1 was achieved by blocking the inhibitory PD-1 signal in the absence of Fc-FcγR engagement, demonstrating that anti-PD1 mAb activity is FcγR-independent (125). By contrast, anti-PD-L1 mAbs displayed significantly enhanced anti-tumour activity when activating FcγR engagement was optimized, effect correlated with the elimination of monocytes and modulation of myeloid cells within the TME. This pathway seems to synergize with the FcγR-independent blocking activity of anti-PD-L1 thereby augmenting the anti-tumour activity of effector T cells (125). Recently, Sow et al. also investigated the effect of the use of different mAbs IgG subclasses on the efficacy of anti-PD-L1 in two mouse models of CRC (126). In the MC38 tumour model, anti-PD-L1 of all IgG subclasses showed similar therapeutic efficacy when compared with each other either in wild-type mice or in mice deficient for all FcγRs. These results are somewhat at odds with the study of Dahan et al. showing a minor enhancement of therapeutic efficacy of anti-PD-L1 mIgG2a over other IgG subclasses in MC38 model. By contrast, in the CT26 tumour model, anti-PD-L1 mIgG2a, showed stronger therapeutic efficacy than other IgG subclasses (126). Recently, Moreno-Vicente et al. have demonstrated that engineered Fc-null anti-PD-1 mAbs are an optimal format to induce effective T-cell anti-tumoral immunity and prevent FcγR-mediated resistance. Preclinical models showed that both anti-PD-1 mIgG1 and mIgG1-N297A, an Fc-null anti-PD-1 mAb, boosted T-cell infiltration, inducing significant and comparable long-term antitumor responses in MC38-bearing mice, whereas anti-PD-1 mIgG2a completely abrogated therapeutic activity (127). Antagonistic anti-CTLA-4 mAbs have been extensively studied in mouse models of cancer. In preclinical models, different studies have shown that anti–CTLA-4 increases the intratumoral CD8+/T reg cell and T eff/T reg cell ratios, promoving preferential depletion of T reg cells at the tumor site (128–130). Highlight that T reg cell depletion is dependent on the presence of Fcγ receptor–expressing macrophages within the tumor microenvironment (130). Thus, anti-CTLA-4 mAbs have revealed that for an optimal therapeutic efficacy binding to Fc receptors for IgG are also required (129, 130). All these findings provide rationale for Fc engineering of these mAbs, using the IgG subclass with the highest affinity to activate FcγR to optimize anti-tumour efficacy.

There is little evidence for the role of specific allotypes of activating FcγR, particularly the FcγRIIIa-V158F, and clinical response in the context of anti-PD-1 or anti-PD-L1 treatment. Nivolumab and pembrolizumab have similar mechanisms of action whereby they competitively inhibit PD-L1 binding by direct occupancy and steric blockade of the PD-L1 binding site (131). Because both present the IgG4 isotype, it only very weakly induces complement and cell activation due to a low affinity for C1q and FcRs (131). Additionally, the heavy chain constant region of nivolumab presents an S228P mutation, which replaces a serine residue in the hinge region with the proline residue found at the corresponding position in IgG1 isotype antibodies. This mutation prevents (Fab´)_2_ arm exchange with endogenous IgG4 antibodies, while retaining the low affinity for activating FcRs associated with wild-type IgG4 antibodies. Therefore, a lack of nivolumab-mediated ADCC or CDC activity is consistent with an expected lack of effector function of IgG4 (132). Anti-PD-L1 IgG1 mAbs inhibit the immunosuppressive PD-L1/PD-1 interaction and may trigger ADCC against cancer cells as an additional anti-tumour activity (133). However, as PD-L1 is also expressed on activated T cells (134), atezolizumab and durvalumab have been engineered with a mutation in the Fc domain to eliminate ADCC and CDC activity, thereby preventing the depletion of T cells expressing PD-L1 (135). In contrast to these mAbs, avelumab is a fully human IgG1 mAb that contains a native Fc region that can bind cognate receptors on immune effector cells and induce ADCC-mediated tumour cell lysis (133). The ability of avelumab to lyse human tumour cells, including lung, breast, and bladder carcinomas in the presence of PBMCs or NK effectors has been demonstrated (136). Recently, Jochems et al. studied the ability of avelumab to increase the lysis of a range of human carcinoma cells by irradiated haNK cells (allogenic NK cell line engineered to express the high affinity (ha) CD16 allele and interleukin-2) *via* ADCC mechanisms (137). Avelumab-mediated ADCC of tumour cells by haNK cells was similar to that of NK cells bearing the V/V Fc receptor high affinity allele (137). Boyerinas et al. demonstrated that healthy donors with FcγRIIIa-158V/V genotype displayed higher avelumab-induced lysis of tumour cells than donors with F/F genotype (136). However, an association between FcγRIIa and FcγRIIIa polymorphisms and PFS in patients with renal cell carcinoma was not found in the molecular analysis performed by JAVELIN Renal 101 trial (n=886) of avelumab plus axitinib vs axitinib (138). Future randomized trials are needed to confirm if the FcγR polymorphisms correlate with a clinical benefit of avelumab.

The association between FcγR polymorphisms and clinical outcome has been also described in response to ipilimumab in patients with advanced melanoma (128). Ipilimumab, a human IgG1 mAb, acts directly on effector T cells by blocking inhibitory signal or through Treg depletion (122). Pre-clinical data in mouse models have demonstrated that the activity of anti-CTLA4 to deplete Treg cells may depend on ADCC (130). These Treg depletions depend on the presence of FcγR-expressing macrophages within TME (130). Recently, Vargas et al. investigated the contribution of Treg cell depletion to the *in vivo* anti-tumour activity of anti-CTLA-4 mAbs in the context of human FcγRs and human IgG isotypes. CTLA-4 was highly expressed by tumour-infiltrating Treg cells in multiple models of transplantable syngeneic tumour cell lines of variable immunogenicity, as well as in human solid tumour subtypes including advanced melanoma, early-stage NSCLC, and renal cell cancer. The expression pattern of activatory FcγRs in human FcγR mice and human tumours was also evaluated. The expression levels of FcγRIIa and FcγRIIIa on innate effector cells appeared higher in the tumour relative to secondary lymphoid organs. Based on these outcomes, the authors determined whether anti-CTLA-4 mAbs of a human isotype promoted depletion of intra-tumoural Treg cells *in vivo*. They observed that anti-CTLA-4 mAbs with the same Fc variants employed in ipilimumab (IgG1) and tremelimumab (IgG2) both induced *in vivo* depletion of tumour infiltrating Treg cells in the context of human FcγRs, mainly in inflamed tumours with high FcγR-expressing innate effector cells (128). This was consistent with the meta-analyses of both Van Allen et al. and Snyder et al., which observed that the high-affinity FcγRIIIa-V158F polymorphism was associated with improved RR in patients with advanced melanoma treated with ipilimumab, but only in the context of high putative neoantigen or indel burden [tumour-specific indel (insertion or deletion) mutations], respectively (p_meta_=0.043 and p_meta_=0.016, respectively) (35, 36). Further, in the Snyder et al. dataset, patients with both high neoantigen burden and the FcγRIIIa-V158F polymorphism had significantly increased OS (p=0.014) (36). These observations were not common to the FcγRIIa-H131R polymorphism, which is associated with greater affinity for IgG2 rather than IgG1. Taken together, these findings suggest that further enhancement of FcγR effector function of anti-CTLA-4 may result in increased anti-tumour activity, and FcγRIIIa-158V/F polymorphism is linked to ipilimumab response in melanoma patients.

## FcR-Dependent Mechanism and Hyperprogressive Disease

On the other hand, several studies indicate that a subset of patients might present accelerated disease progression and clinical deterioration on treatment with anti-PD-1/anti-PD-L1 mAbs (139). This setting is referred to as hyperprogressive disease (HPD), which is a new outcome pattern with as yet unknown biological mechanisms. Recent studies in this issue point to a possible role of FcRs in this process (140, 141). In immune-deficient mice, PD-1 blockade accelerated growth of M109 PD-1 knockout-xenograft tumours with increased proliferation and decreased apoptosis (140). In similar xenograft models, Lo Russo et al. tested the F(ab)_2_ moiety of nivolumab in comparison with whole Ab and showed that this anti-PD-1 without the Fc domain no longer induces HP-like disease in tumour-bearing athymic mice (119). This research suggested a possible role for FcγRIIb in the detrimental effect associated with anti-PD-1 therapy, and concluded that this phenomenon is maintained by myeloid cells, such as M2-like tumour-associated macrophages (TAM), within the TME by FcR-dependent mechanisms probably through inhibitory receptors (141). Further studies elucidating the involvement of FcRs in the development of HPD are required.

## Fc-Optimization of Therapeutic Monoclonal Antibodies

Optimized FcR-mediated effector functions in order to improve clinical efficacy and safety of the immunotherapy have been addressed with modification strategies such as altered glycosylation patterns, point mutations, combination of different Fc subclasses (cross isotypes), and Fc-truncation of the mAbs (142).

Diverse mAbs targeting various antigens have been modulated by N-linked glycosylation in the Fc region of the antibody (143). Absence of core fucose from the Fc N-glycan has demonstrated an enhanced IgG1 binding affinity specifically for FcγRIIIa, resulting in increased ADCC/ADCP effector function both *in vitro* and *in vivo* (144). Thus, modification of Fc-FcγR interactions through glycoengineering of the Fc N-glycan at Asn297 of the CH2 domains is a relevant strategy to produce afucosylated therapeutic mAbs (reviewed in (5, 145). Fc glyco-engineering antibodies are being assessed and some have currently received approval for clinical use in haematological disorders (see below) (146). However, the afucosylated mAbs developed in solid tumours have not achieved the expected results in clinical trials. As an example, tomuzotuximab, an afucosylated EGFR-directed mAb, failed to demonstrate improved efficacy compared to cetuximab in the first-line treatment of recurrent or metastatic HNSCC (RESGEX study) (147).

Another approach to increase affinity in the antibody-FcγR interaction and, therefore, the *in vivo* activity of these therapeutic mAbs, includes engineering the Fc region through amino acid mutations. Several IgG subclass mutations have been described that affect Fc-FcγRs interactions (9). For example, some mutations in IgG1, such as N297A and L234A/L235A, or S239D/H268F/S324T/I332E, reduce/abrogate or increase binding to all FcγRs, respectively. Therefore, these genetic variants can provoke alterations in the ADCC mechanism carried out by mAbs (9). Different groups have examined the introduction of a point mutation in the IgG Fc domain aiming to increase the effector function of FcγR-expressing cells (148–154). Ashoor et al. developed a tool-box of IgG1 Fc isoforms to depict the affinity between mutated IgG1 Fc regions and FcγRIIIa-V158F variants (149). The investigators designed, cloned and expressed human extracellular domains of FcγRIIIa-V158F and six different upper hinge mutated isoforms (M1-M5 and M10) of human IgG1 Fc moiety (Hinge, CH2 and CH3 chains) in *Pichia pastoris*. The Surface Plasmon Resonance method along with an *in silico* analysis showed that mutation M1 and M2 had higher affinities to the low binding allele of FcγRIIIa (158F/F) than wild type Fc (3.3-fold and 2.7 fold, respectively) and, in addition, demonstrated that the affinity of the Fc region to the FcγRIIIa is strongly correlated with polar interactions. They concluded that this molecular engineering approach allows for the generation of therapeutic mAbs endowed with high ADCC function (149).

In this context, Margetuximab is an Fc-engineered ERBB2-targeted antibody that shares epitope specificity and Fc-independent antiproliferative effect with trastuzumab (153). However, with a five amino acid substitutions engineered into the margetuximab IgG1 Fc domain (L235V/F243L/R292P/Y300L/P396L) it has been obtained an increased binding affinity for both allelic variants of the low-affinity activating FcγRIIIa and decreased affinity for the inhibitory FcγRIIb, resulting in improved effector functions, such as ADCC (153, 155). Recently, based on the results of the phase III SOPHIA (n=536) trial, margetuximab has been approved for use in combination with chemotherapy as treatment in previously-treated metastatic HER2-positive BC (156). In this trial, margetuximab plus chemotherapy had a statistically significant improvement in PFS over control in the whole-study population. In addition, treatment effects were more pronounced in patients with the FcγRIIIa-158F allele, corresponding to 437 of the 506 patients’ genotype (86%). Among these patients, the median PFS was 6.9 months with margetuximab versus 5.1 months with trastuzumab (HR=0.68, 95% CI, 0.52-0.90; p=0.005). Conversely, there was no margetuximab benefit over trastuzumab in the smaller FcγRIIIa-158V/V group (24).

Enoblituzumab (MGA271) is a humanized B7-H3 mAb that incorporates Fc-domain modifications (L235, F243L, R292P, Y300L, and P396L) designed to enhance antitumor effector-mediated function. B7-H3 is a protein in the B7 family of immune regulator proteins. B7-H3 is widely expressed by a number of different tumour types and may play a key role in regulating the immune response to various types of cancer. In several mouse cancer models, ectopic expression of B7-H3 has been shown to lead to activation of tumour-specific cytotoxic T cells that can slow tumour growth or even completely eradicate tumours. However, B7-H3 also acts as a T cell coinhibitor, inhibiting CD4 T cell activation and NK cell function through unidentified receptor(s), and the production of effector cytokines such as IFN-γ and IL-4 (157). In human CD16a-bearing transgenic mice, enoblituzumab exhibited potent antitumor activity in B7-H3–expressing xenograft models of renal cell and bladder carcinoma (152). In a Phase 1 clinical study, Enoblituzumab was evaluated in combination with an anti-PD-1 mAb in patients (n=133) with B7-H3-expressing melanoma, HNSCC, NSCLC and UC. In the HNSCC dose expansion cohort, ORR occurred in 6/18 (33%) response-evaluable, checkpoint-inhibitor-naïve HNSCC patients, including 4 confirmed and 2 unconfirmed PR, with SD in 6/18 (33%). In NSCLC patients (PD-1 naïve, tumour PD-L1 <1%), there were 4/14 PR (29%) and 9 SD (64%). Two of 16 post-checkpoint-inhibitor UC patients achieved a PR and unconfirmed CR, respectively (158). Several clinical trials with enoblituzumab in patients with different solid tumour are ongoing (NCT02923180, NCT02475213, NCT02381314, NCT02982941).

Other variants containing amino-acid exchanges, such as S239D/I332E (SDIE modification), to enhance affinity to FcγRIIIa on NK cells have been evaluated in experimental models (151, 154). For example, Raab et al. reported an Fc-optimized NKG2D-Fc construct, carrying the SDIE modification, which enhanced degranulation, ADCC, and IFN-γ production of NK cells in response to BC cells, independently of FcγRIIIa-V158F polymorphism (154).

In hematologic neoplasms, a new generation of Fc-engineered CD20-targeted mAbs has emerged for the treatment of B-cell malignancies and autoimmune disease (159). These novel anti-CD20 mAbs can be effective at activating innate immune cytotoxic mechanisms, including CDC, ADCC and ADCP (160). The anti CD20 Fc regions have been manipulated by defucosylation (e.g. obinituzumab) (161) or amino acid engineering (e.g. ocaratuzumab) to enhance FcR affinity. Pre-clinical studies with ocaratuzumab have shown an advantage in NK cell-mediated ADCC over other CD20 mAbs (rituximab or ofatumumab) (150). Cheney et al. reported that low concentrations (0.1-10 µg/mL) of ocaratuzumab improve allogenic and autologous ADCC ~3-fold and ~1.5-fold compared with rituximab or ofatumumab against CLL cells, respectively (162). More recently, in similar experiments, VanDerMeid et al. demonstrated the important contribution of ocaratuzumab and ofatumumab in ADCP (160). These authors showed that CD20 mAbs induce an ADCP >10-fold more cytotoxic than ADCC in a primary human cell model. However, Fc engineering also abrogated complement activation, an important mechanism for activating ADCP. Moreover, ocaratuzumab and ofatumumab induced ADCP at 10-fold lower concentrations than rituximab. CD20 mAb-induced ADCP was not inhibited by venetoclax and was less inhibited by Bruton´s tyrosine kinase inhibitors and PI3Kδ inhibitors (160). These data suggest the need to test a wide-range of doses and intervals of administration to establish optimal next-generation anti-CD20 therapeutic regimens.

In the clinical setting, safety and efficacy of ocaratuzumab in fifty previously treated FL patients with a low-affinity genotype of FcγRIIIa have been evaluated with an investigator-assessed RR of 30% (15/50), including four CR, three unconfirmed CR and eight PR, and a median PFS of 38.3 weeks (163). This study suggests that ocaratuzumab might be more effective in FcγRIIIa-158F-carriers. Further studies are needed to establish the role of ocaratuzumab in the treatment of patients with CD20+ FL.

Two Fc-optimized anti-CD19 mAbs have been developed for the treatment of paediatric B-linage acute lymphoblastic leukaemia (ALL), inebilizumab (modifying the Fc-linked N-glycan) and XmAb^®^5574 (a version of 4G7-anitbody with SDIE modification in the human Fc-domain of IgG1) (164). These antibodies have shown enhanced potency ADCC and ADCP compared to the unmodified IgG1 CD19 antibody, as demonstrated by *in vitro* and *in vivo* analyses in leukaemia and lymphoma animal models [reviewed in (165)]. XmAb^®^5574 has been demonstrated to be safe and to have some efficacy in patients with relapsed CLL (166). The antitumor activity and safety of XmAb^®^5574 were also evaluated in patients with r/r B-NHL, including DLBCL (n=35), FL (n=34), other indolent NHL (iNHL; n=11) and MCL (n=12) (167). ORR, including CR, were seen in 26% of patients with DLBCL, 29% with FL and 27% with iNHL. They lasted ≥12 months in 5/9 responding patients with DLBCL, 4/9 with FL and 2/3 with other iNHL, showing a median duration of 20.1 months for DLBCL, not yet reached for FL and other iNHL. In conclusion, XmAb^®^5574 is an alternative CD19-targeted agent that improves outcomes in r/r B-NHL patients. XmAb^®^5574 is currently being evaluated in a clinical trial with adult B-ALL patients (NCT01685021).

Compared with other FcγR, the inhibitory role of FcγRIIb may be disadvantageous to antibody-based therapies and other immune stimulating therapies. Pre-clinical models have shown that upregulation of the FcγRIIb at the tumour site prevented intra-tumoral Treg cell depletion carried out by anti-CD25 mAb, which limits its activity against established tumours (148). This lack of therapeutic activity can be reversed through the use of an Fc-optimized anti-CD25 mAb. Vargas et al. demonstrated that use of an anti-CD25 antibody with enhanced binding to activating FcγRs led to effective depletion of tumour-infiltrating Treg cells, increased effector to Treg cell ratios, and improved control of established tumours (148). This variant of Fc-optimized anti-CD25 could be a promising therapeutic strategy in combination with novel immunotherapies (e.g. ICIs).

FcR-blocking antibodies are other novel strategies to enhance therapeutic antibody efficacy. As pointed out above, FcγRIIb promotes anti-CD20 mAb internalization and confers therapeutic resistance to rituximab in B cell lymphoma (68). Anti-FcγRIIb antibodies to prevent FcγRIIb-mediated CD20-rituximab internalization have been elaborated (69). In patient-derived xenograft models, BI-1206, an FcγRIIb antagonistic antibody, the efficacy of rituximab-based therapies in aggressive mantle cell lymphoma was improved (168). FcγRIIb blocking mAb are being developed in the clinic to overcome rituximab resistance (NCT03571568), enhance anti-PD-1 activity (NCT04219254), and enhance anti-Her2 activity (EudraCT Number: 2021-005646-15).

Finally, although the biological consequences of SNPs in high-affinity FcγRs (e.g. V39I or I338T) and other low-affinity FcγRs, such as the FcγRIIb-I232T polymorphism, have been described, the role of these genetic variants in mAb immunotherapy has not been well established (78, 106). This is probably due to the low frequency of these polymorphisms in the population (96).

## Concluding Remarks

Targeted therapy with mAbs have generated a high expectation of success against cancer. However, since not all patients respond to mAbs, optimal selection of patients for treatment is essential to avoid therapeutic failure. Different functional FcγR polymorphisms have been postulated as biomarkers of therapeutic response in a widespread variety of tumours. The above-mentioned studies highlight the potential relevance of FcγR polymorphisms for NK effector function mediated through ADCC activity and their potential clinical impact.

Despite some variability among studies, a general trend to clinical benefit has been observed in patients whose cells express high affinity FcγRIIa/IIIa genotypes treated with IgG1 mAbs. The conflicting results may be due to multiple factors, including the difficulty to detect the FcγRIIIa-158V/V allele due to high homology with FcγRIIIb, the amount of antibody ligand occupied necessary for mediating an effective ADCC on tumour cells, differences in therapeutic scheme, as well as inter-patient and intra-tumoral heterogeneity (137). Theoretically, an unfavourable FcγR genotype cannot reasonably be used to exclude candidates for mAb therapy, since their activity can also be exerted by Fc-independent mechanisms. Other predictive biomarkers could be jointly evaluated in mAb-based treatment. Moreover, a limitation of previous studies is the fact that FcγR CNVs are not included as a possible association variable. Future studies should include or consider genotyping FcγR CNVs as effects of FcR CNVs on immune responses are more profound and have been demonstrated in numerous association studies between FcγR CNVs and autoimmune inflammatory diseases.

Novel advances in the role of FcγR-binding on ICI therapy have been elucidated. ICIs can modulate the tumour immune microenvironment through FcγR-dependent mechanisms, as mentioned for anti-CTLA-4 mAbs and depletion of Treg, or anti PD-L1 avelumab and myeloid subset composition. Therefore, additional studies are required to precisely define the role of FcγR polymorphisms in the clinical outcome of therapy with ICIs.

In summary, these insights into FcγR polymorphisms might allow the development of more effective mAbs in the field of cancer therapy.

## Author Contributions

All authors contributed to the conceptualization, writing, editing and table development. All authors contributed to the article and approved the submitted version.

## Conflict of Interest

The authors declare that the research was conducted in the absence of any commercial or financial relationships that could be construed as a potential conflict of interest.

## Publisher’s Note

All claims expressed in this article are solely those of the authors and do not necessarily represent those of their affiliated organizations, or those of the publisher, the editors and the reviewers. Any product that may be evaluated in this article, or claim that may be made by its manufacturer, is not guaranteed or endorsed by the publisher.
